# Effects of fatigue on motor unit characteristics in isometric elbow contractions across age groups

**DOI:** 10.3389/fnins.2025.1747360

**Published:** 2026-01-05

**Authors:** Huaning Kuang, Fang Qiu, Xiaodong Liu, Chen Chen

**Affiliations:** 1 State Key Laboratory of Mechanical System and Vibration, Shanghai Jiao Tong University, Shanghai, China; 2 School of Physical Education and Health, Shanghai University of International Business and Economics, Shanghai, China; 3 Institute of Physical Education, Guangxi University of Science and Technology, Liuzhou, China

**Keywords:** aging, discharge property, fatigue, high-density surface electromyography, motor unit, neural drive

## Abstract

This study aims to investigate the neuromuscular regulation mechanisms in different age groups under conditions of fatigue. A total of 50 participants aged between 10 and 80 years were recruited and divided into 11 different age groups. Each participant performed a triangular wave isometric contraction before and after fatigue. The convolution kernel compensation algorithm was employed to extract the motor unit (MU) spike trains from the biceps brachii and triceps brachii muscles. The extracted features included root mean square, mean power frequency, motor unit action potential (MUAP) peak-to-peak values, MUAP duration, recruitment threshold, derecruitment threshold, discharge frequency, and common neural drive, which were compared across age groups before and after fatigue. Significant variations in fatigue adaptation were observed among age groups, according to the study. Adults tend to recruit larger motor units to compensate for fatigue-induced strength decline. The elderly group’s discharge frequency and MU recruitment threshold, on the other hand, did not significantly change, indicating that their neuromuscular system was less able to adjust to fatigue. The child group may have relied more on discharge frequency modulation during fatigue because they displayed smaller variations in recruitment threshold. At the same time, PCA results showed that fatigue induced a greater reduction in common-drive coherence in children and older adults, suggesting that maintaining coordinated neural drive becomes more challenging during development and aging. This study clarifies the differences in neuromuscular fatigue adaptation mechanisms at different stages of life and reveals the changing patterns of MU regulation strategies across the examined age range. The resulting age-dependent MU patterns provide an important physiological reference for neurological healthcare, facilitating clearer identification and interpretation of deviations associated with neuromuscular and neurological disorders.

## 1 Introduction

Existing studies have shown that during muscle fatigue caused by sustained contractions, the central nervous system compensates through two primary mechanisms: (1) adjusting the recruitment strategy of motor units (MUs), and (2) changing the discharge frequency of MUs. These adaptive changes are designed to counteract the decrease in muscle force-generating capacity resulting from fatigue (McManus et al., 2015; Mota et al., 2020). Valenčič et al. (2024) found in their study of fatiguing isometric knee extension tasks to failure that MU discharge rate changes are influenced by both contraction modality and intensity. In low-intensity sustained contractions, discharge rate decreases initially and then returns to baseline, while in higher-intensity or intermittent tasks, discharge rate progressively increases with fatigue in the latter stages of the task. Angius et al. (2024) studied fatiguing isometric contractions of the knee extensors and found that high-intensity exercise (70% MVC) caused greater force decline and increased excitability in both corticospinal and spinal pathways, while motor unit discharge rate increased in both low- and high-intensity tasks, indicating compensatory neural adjustments to fatigue. Meanwhile, fatigue also reshapes MU functional characteristics; under isometric conditions, Liu et al. observed increased MU synchronization in an upper-limb biceps brachii task (Liu et al., 2021). Additionally, fatigue may induce morphological changes in MUs (Jesunathadas et al., 2010).

Although extensive research has been conducted on fatigue (Cifrek et al., 2009; Jesunathadas et al., 2010; Jones et al., 2023), studies on the effects of aging on fatigue remain relatively limited (Jordan et al., 2013; Pascoe et al., 2013). There are significant differences in the neuromuscular systems across different age groups, including MU count, muscle fiber type composition, and neural drive patterns, which may result in different fatigue adaptation mechanisms (Merletti et al., 2002). Research has suggested that the neuromuscular system of children is not yet fully mature, which exhibits poorer MU discharge stability and less differentiated recruitment hierarchies, as observed during isometric finger abduction of the first dorsal interosseous in both males and females (Miller et al., 2019). Adult men typically exhibit optimal neuromuscular control than older men during isometric plantar flexion of the soleus (Kallio et al., 2012). By contrast, studies have reported a gradual decline in neuromuscular function in elderly adults, including a reduction in MU count (Kedlian et al., 2024), loss of *α*-motor neurons (Dalton et al., 2010; Power et al., 2014), selective atrophy of Type II fibers, and decreased neuromuscular junction function (Mitchell et al., 2012; Hunter et al., 2016). These factors collectively lead to a decrease in muscle strength and a reduction in motor control precision in elderly adults (Hu et al., 2021).

While some studies have investigated the effects of aging on fatigue adaptations, most focus only on the differences between young and older adults, with a lack of systematic comparisons across other age groups (Paris et al., 2022). For example, research by Rubinstein and Kamen (2005) on the tibialis anterior during repeated sustained maximal isometric dorsiflexion contractions in women suggests that older adults may exhibit greater resistance to fatigue because younger adults experience a greater decrease in motor unit discharge rate during fatigue than older adults. Moreover, during isometric elbow flexion of the biceps brachii, fatigue not only affects the recruitment and derecruitment strategies of MUs but may also alter the synergistic effect of neural drive in both males and females (He et al., 2023). Although common input is essential for coordinating muscle contractions because it improves force output and stability by synchronizing the firing of multiple MUs, few studies have used principal component analysis (PCA) to quantify the differences in common neural drive before and after fatigue across different age groups (Farina et al., 2014; Benamati et al., 2024). We chose PCA because it effectively reduces dimensionality while retaining key variations in high-dimensional data, allowing us to analyze neural drive and motor unit activity essential for understanding fatigue adaptations across age groups.

High-density surface electromyography (sEMG), with its outstanding spatial resolution, provides a novel technical approach for non-invasive research into the mechanisms of motor nerve control (Geng et al., 2022). By combining it with blind source separation algorithms such as convolution kernel compensation (CKC), it is possible to accurately extract motor unit spike trains (MUSTs) from high-density sEMG recordings (Negro et al., 2016). Based on this technological advantage, this study aims to conduct a preliminary investigation of age-related differences in MU recruitment strategies, motor unit action potential (MUAP) morphology, and neural drive during fatigue across three broad age categories using high-density sEMG.

Guided by prior work showing that motor control is optimal in adulthood, immature in children, and declines with aging, we hypothesize that fatigue-induced changes in MU and sEMG indices will differ across age groups. Specifically, adults will exhibit the lowest force-tracking error, whereas children and older adults will show higher root mean square error (RMSE). Fatigue will increase RMSE in all groups, with larger increases in older adults. For sEMG, mean power frequency (MPF) will decrease and root mean square (RMS) will increase after fatigue, with the magnitude of change differing by age. At the motor-unit level, fatigue is primarily compensated for by recruiting higher-threshold MUs in adults, while children rely more on adjustments in discharge rate, and older adults exhibit blunted compensatory adjustments. Finally, we expect the common neural drive to be maintained or enhanced in adults but reduced in children and older adults under fatigue.

## 2 Methods

### 2.1 Subjects

**Figure 1:**
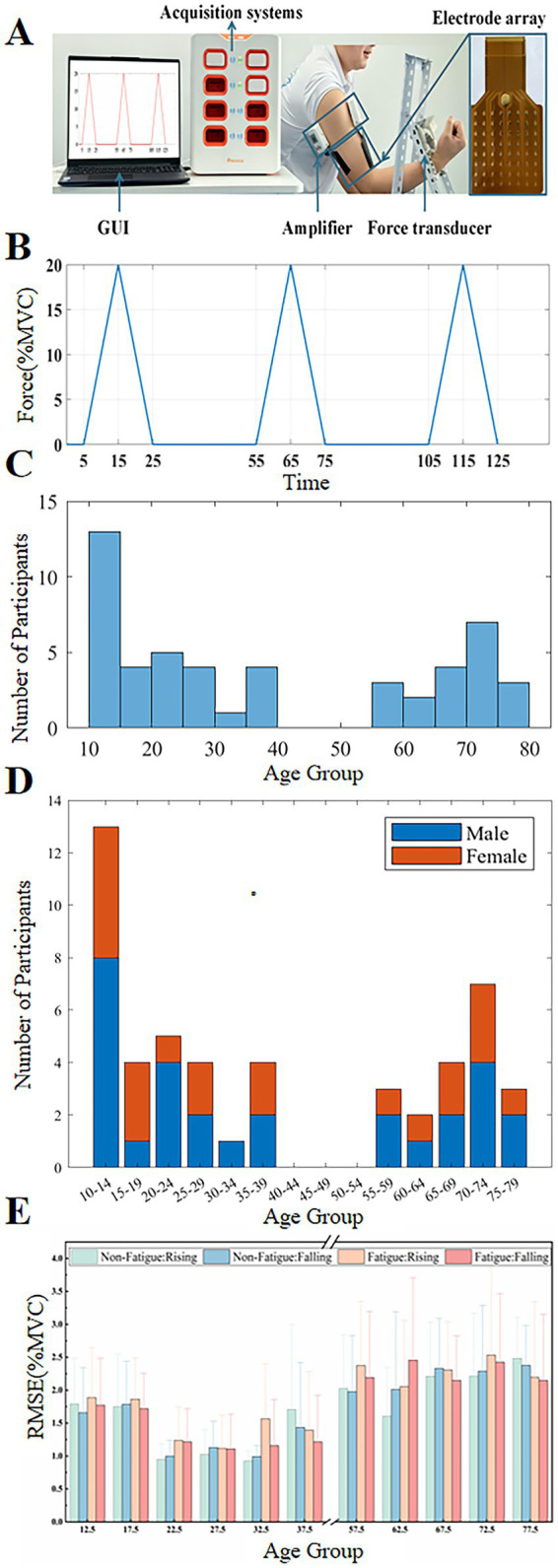
Experiment and force tracking performance. **(A)** Experimental setup. **(B)** Target curve. **(C)** The number of subjects in each age group. **(D)** Displays the gender composition by age group. **(E)** The RMSE of force during the rising and falling phases of the triangular wave force trajectory before and after fatigue across age groups.

Fifty subjects (29 males and 21 females, aged 10–80 years) were recruited for the experiment, who were habitually right-handed. The subjects were divided into 11 groups according to age, with intervals of 5 years. The specific groupings and the number of participants in each group are shown in Figures 1C,D displays the gender composition by age group. The 40–55 age group is missing, and there is only one participant in the 30–35 age group. For clarity, we refer to participants aged 10–20 years as the child group, 20–40 years as the adult group, and 55–80 years as the elderly group throughout the manuscript. All subjects had no history of neuromuscular disease and had not engaged in any strenuous exercise within 24 h before the start of the experiment. All subjects were informed about the experimental procedures and signed an informed consent form before participating in the experiment. The experimental protocol and informed consent process adhered to the Declaration of Helsinki and received approval from the local ethics committee of Shanghai Jiao Tong University (approval number E20240248I).

### 2.2 Experiment

#### 2.2.1 Experimental settings

In this study, a customized experimental platform was constructed for the simultaneous acquisition of force signals and high-density sEMG signals during an isometric elbow flexion task. A force transducer (Hand Grip Dynamometer, Biometrics Ltd., UK) mounted on a stand was in constant contact with the middle of the forearm of subjects who were comfortably seated in front of the platform. Their dominant arm was extended forward with an angle of about 100° maintained between the upper arm and forearm (Figure 1A). To minimize off-target movements, the elbow was immobilized by strapping, and subjects were asked to activate the upper arm muscle groups primarily during force application. The force transducer was calibrated prior to each session. Within-subject reproducibility was evaluated by comparing the repeated trials. Force signals were acquired in real-time at a sampling rate of 200 Hz through a MATLAB-developed graphical user interface, which collected data and provided visual feedback to the subject via serial communication.

Meanwhile, sEMG signals were acquired at the muscle bellies of the biceps and triceps using a 64-channel high-density wet electrode array (8 × 8 arrangement, Grid8X8P-8 mm, Onesense, China). Each electrode in the electrode array had a diameter of 3 mm and was spaced 8 mm apart in both directions. The electrode array was coupled to a multichannel acquisition module. sEMG signals were transmitted wirelessly to a laptop computer with a gain of 1,000 Hz and a sampling rate of 2,000 Hz. The sEMG signal was filtered through hardware with a bandpass range of 3 Hz to 900 Hz and then converted into a digital signal using a 24-bit analog-to-digital converter. To ensure the synchronization of the force signal with the sEMG signal, an Arduino-based custom synchronization module was used to generate trigger signals at the beginning and end of each data acquisition.

#### 2.2.2 Experimental protocol

This study consisted of three experimental sessions: (1) maximum voluntary contraction (MVC) measurement, (2) muscle force and high-density sEMG signal acquisition in the non-fatigue state, and (3) muscle force and high-density sEMG signal acquisition in the fatigue state. Throughout the experiment, subjects were required to perform an isometric elbow flexion task.

Before the start of the formal experiment, all subjects were required to perform the MVC test. The average of the maximum force of the three contractions was defined as the MVC force value for that subject. In the data acquisition phase of the non-fatigue state, the experiment was guided by visual feedback. The force sensor recorded the contraction force applied by the subject in real-time, while the upper computer interface displayed the target contraction force curve and the actual contraction force curve. We used a triangular isometric force trajectory to estimate MU behavior during both recruitment and derecruitment within the same trial. This dynamic approach allowed us to capture real-time adjustments in the MU pool, offering insights into MU activation patterns that a steady plateau could not. The subject must try their best to make their force curve consistent with the target curve. A triangle wave with a peak value of 20% MVC and a rise and fall rate of 2% MVC/s was the target curve (Figure 1B). Each subject repeated the triangular wave task three times, with a 30-s break between tasks in the non-fatigue state. Participants then performed sustained isometric contractions at 30% of maximal voluntary contraction until fatigue (Wang et al., 2021). Using a common fatigue criterion to compare motor unit changes across age groups is a standard method for identifying age-related neuromuscular differences (Dos Santos et al., 2020; Mota et al., 2020). Fatigue was defined as either the force trace falling below the 30% target and not being promptly restored despite standardized verbal encouragement or the participant reporting an inability to continue, at which point the trial was terminated. After fatigue induction, the subjects immediately performed the same triangular wave force task as in the non-fatigued state.

### 2.3 Data analysis

#### 2.3.1 Preprocessing

For the force signals, the data were first up-sampled to 2,000 Hz, after which a 2.5 Hz low-pass filter was used to reduce high-frequency noise. A 4th-order Butterworth filter was applied to the sEMG signals, with a processing frequency range of 20 Hz to 500 Hz. A 50 Hz comb filter was also added to remove power disturbances. Channels with abnormal amplitude were removed, usually fewer than five, possibly due to poor electrode contact.

#### 2.3.2 sEMG signal decomposition

The sEMG signals were decomposed using the CKC algorithm and an iterative estimation algorithm based on the natural gradient descent algorithm to extract the MUSTs (Holobar and Zazula, 2004; Holobar and Zazula, 2007b). Briefly, this is done by expanding the sEMG signal and estimating the MUST as:


$${\hat{s}}_{j}(n)=c_{s_{j}\overset{-}{x}}^{T}C_{\overset{-}{x}\overset{-}{x}}^{-1}\overset{-}{x}(n)$$

Where $C_{\overset{-}{x}\overset{-}{x}}=E(\overset{-}{x}(n){\overset{-}{x}}^{T}(n))\;$ is the covariance matrix of sEMG signals, and $c_{s_{j}\overset{-}{x}}=E(\overset{-}{x}(n)s_{j}^{T}(n))$ contains the cross-correlation vector between each pulse train and sEMG signals, E denotes the mathematical expectation.

In this study, a natural gradient descent algorithm was used to iteratively estimate the cross-correlation vectors ($c_{s_{j}\overset{-}{x}}$) to extract the MUSTs (Holobar and Zazula, 2007a). Three replicate trials were performed before and after fatigue. The separation matrix ($c_{s_{j}\overset{-}{x}}^{T}C_{\overset{-}{x}\overset{-}{x}}^{-1}$) from each trial was kept for decomposing the signals from the other two trials. The MUSTs for that trial were obtained by combining and filtering the MUSTs identified in the separation matrices of the three trials. The pulse-to-noise ratio (PNR) was used to measure the difference between the baseline noise and the MUAP discharge pulse (Holobar et al., 2014). In this study, MUSTs with a PNR below 25 dB or a discharge frequency above 35 Hz were excluded. These methods ensured reliable motor-unit identification. However, we acknowledge that not all motor units, particularly those in deeper muscles, could be successfully tracked. This limitation was described in detail in the Limitations section of the manuscript. Given that fatigue commonly shifts the active motor-unit pool, we did not explicitly match individual motor units from pre- to post-fatigue. Instead, we assessed global neuromuscular adaptations using aggregate changes across all identified motor units within each age group. Multichannel MUAP waveforms for each MU were obtained by spike-trigger averaging (Lodha et al., 2010). Taking the discharge timings of each MU as the trigger point, the sEMG signals before and after were intercepted for 64 ms. The MUAP waveforms were then obtained by averaging the signals at various discharge moments. The waveforms of all channels were extracted to obtain the MUAP waveform array.

#### 2.3.3 Feature extraction

As summarized in Table 1, the key features extracted in this study are outlined, providing a comprehensive overview of the neuromuscular characteristics analyzed. The RMSE of the force signals was extracted to assess force tracking performance in different age groups (Ballardini et al., 2019). To characterize the changes of MUs in the time-frequency domain features, RMS and MPF were extracted for the biceps and triceps brachii muscles before and after fatigue, respectively, for the ascending and descending phases of the triangle wave force trajectory.

**Table 1:** Summary of extracted features.

Feature category	Extracted feature	Abbreviation
Time-frequency features	Root mean square	RMS
	Mean power frequency	MPF
MUAP morphology	Motor unit action potential peak-to-peak value	MUAP PPV
	Motor unit action potential duration	MUAP duration
Discharge properties	Discharge rate	DR
	Recruitment threshold	RT
	Derecruitment threshold	DT
Common neural drive	PC1 variance explained	PC1

To characterize MUAP changes, this study extracted the maximum peak-to-peak value (PPV) of the MUAP waveform across all channels and the duration for the corresponding channel. The PPV was defined as the voltage difference between the maximum peak value and the adjacent minimum peak value of the waveform within a time window. The duration, on the other hand, was defined as the time interval between the maximum and minimum values in the MUAP waveform.

Detailed statistical analyses were performed to assess changes in MU recruitment and derecruitment thresholds, as well as the mean discharge rate in different age groups. After integrating the muscle force signals and MUSTs, the recruitment threshold was defined as the level of muscle force required for the appearance of the first MUAP. In contrast, the derecruitment threshold was the level of force at which the MU ceased to contribute to the generation of muscle force during force reduction (Del Vecchio et al., 2020a). In the rising phase, we took the first five action potentials after the motor unit began firing, calculated the mean inter-spike interval (ISI), and used its inverse as the motor-unit discharge rate. The MVC corresponding to the MU discharge rate in the rising phase represents the recruitment threshold. In the falling phase, we averaged the ISIs of the last five spikes in the train and took the inverse to obtain the discharge rate during derecruitment. The MVC corresponding to this MU discharge rate in the falling phase represents the derecruitment threshold.

Finally, we analyzed the common driving information of the MUs (Holobar and Glaser, 2019). The discharge rate curve for each MU was calculated using the MUST signal of each trial, and the resulting data were subsequently subjected to PCA. In this analysis, the variable dimension was equal to the number of MUs, and the number of test sampling points determined the sample size. We focused on these three metrics before and after fatigue as follows: (1) the proportion of variance explained by the first principal component (PC1) of the eigenspace, (2) the Pearson correlation coefficient between PC1 and the muscle contractility curve, and (3) the Pearson correlation coefficient between PC1 and the cumulative spike train (CST). The MUSTs of all MUs in the trial were combined and sorted chronologically to form a unified spike train, and the discharge rate of this spike train was defined as the CST.

To assess the changes in the characteristics of different age groups after the fatigue, this study used relative change ratio (RCR) as a quantitative index, which was calculated as:


$$\mathrm{RCR}=\frac{\text{Fatigue value}-\text{Non}-\text{fatigue value}}{\text{Non}-\text{fatigue value}\;}\times 100\%$$

This method eliminated baseline differences through standardization, making comparisons of fatigue effects among age groups comparable.

### 2.4 Statistics

To comprehensively assess the effects of fatigue and age, we performed a mixed 2 (condition: non-fatigued, fatigued) × 10 (age group) ANOVA on the MU characteristics. This primary analysis allowed us to explicitly test and report the main effects of fatigue and age, as well as their interaction. When Shapiro–Wilk tests revealed non-normal distribution, data were log-transformed for further comparisons using ANOVA. Partial eta-squared ($\eta_{p}^{2}$) was reported as the measure of effect size. If interactions were significant, post-hoc comparisons for each factor were made, and the level of significance was adjusted for multiple comparisons by using Bonferroni correction. All analyses were performed in SPSS (Version 25, IBM, Armonk, NY, USA) and MATLAB R2021b (MathWorks, USA).

In addition, to directly quantify and compare the magnitude of fatigue-induced adaptation across age groups, which is the central focus of our research question, we also conducted a one-way ANOVA on the relative change ratio. Before conducting the one-way ANOVA, data were tested for normality and homogeneity of variances. Post-hoc analyses were conducted using Bonferroni when variances were aligned; otherwise, Dunnett’s C test was applied (Dunnett, 1955). The significance level was set at 0.05. If the difference between the two groups was not significant (*p* > 0.05), the same letter was shared. If the difference was significant (*p* ≤ 0.05), different letters were labeled.

## 3 Results

### 3.1 Force tracking performance and time-frequency domain characterization of sEMG

The distinctive pattern of RMSE with age in the force tracking task, both before and after fatigue, is displayed in Figure 1E. As shown in the figure, the adult group (20–40 years old) exhibited the best force tracking performance, with a lower RMSE compared to the other age groups. Both the child group (10–20 years old) and the elderly group (55–80 years old) showed poor force tracking performance, with the elderly group having the largest RMSE, indicating that the elderly group had less control over force (Weavil et al., 2018). In fatigue, RMSE showed a certain increasing trend in all age groups. This phenomenon was particularly evident in the 55–65 age group.

**Figure 2:**
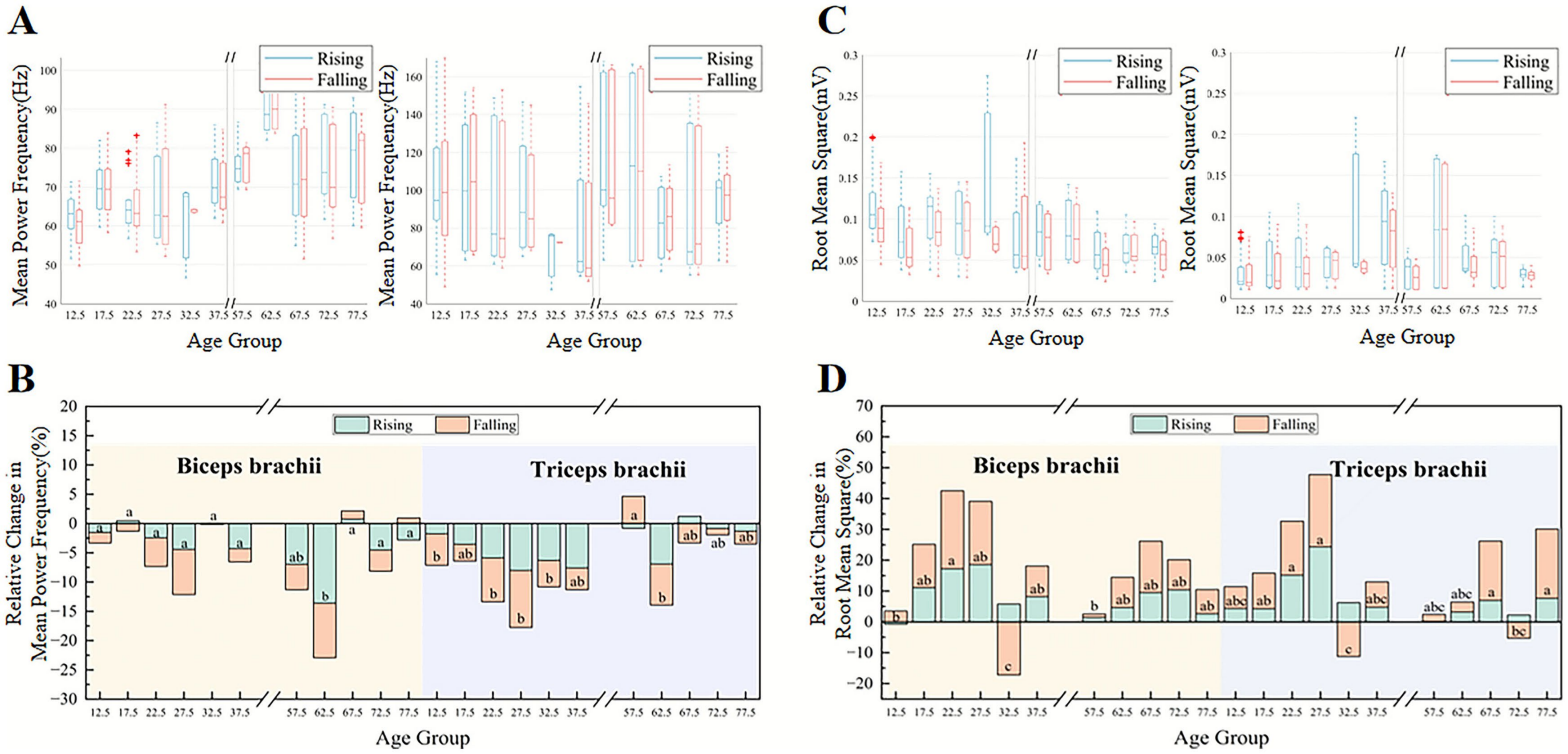
The changes in RMS and MPF before and after fatigue across different age groups. **(A,C)** The MPF and RMS of the sEMG signals of the biceps (left) and triceps (right) across age groups before fatigue in the triangular wave force trajectory, while **(B,D)** show the relative changes in MPF and RMS after fatigue.

Figure 2 shows the time-frequency domain characteristics of the sEMG signals of the biceps brachii and triceps brachii before and after fatigue across different age groups. In the biceps brachii, the MPF during the rising phase was significantly affected by fatigue, age, and their interaction (fatigue: *F*(1,39) = 21.94, *p* < 0.001, $\eta_{p}^{2}$=0.36; age: *F*(9,39) = 3.10, *p* = 0.007, $\eta_{p}^{2}$=0.417; interaction: *F*(9,39) = 4.15, *p* < 0.001, $\eta_{p}^{2}$=0.49). As shown in Figure 2A, in the adult group (20–40 years old), the MPF of the biceps brachii during the force falling phase was significantly lower than that during the rising phase. In contrast, the most elderly groups (55–80 years old) exhibited an opposite pattern, with the MPF in the falling phase generally higher than in the rising phase. Additionally, the MPF of the biceps brachii was significantly lower in the child group (10–15 years old) than in the elderly group (55–80 years old). Figure 2B shows the relative change in MPF after fatigue. After fatigue, most age groups exhibited a decline in MPF due to the increase in low-frequency components. In the force increase phase, the 55–65 age group showed the most significant decrease in the relative change in MPF of the biceps brachii.

Figure 2C displays the RMS of the biceps brachii and triceps brachii before fatigue in each age group. In the biceps brachii, the RMS during the falling phase was significantly affected by fatigue, age, and their interaction (fatigue: *F*(1,39) = 6.277, *p* = 0.017, $\eta_{p}^{2}$=0.139; age: *F*(9,39) = 3.61, *p* = 0.002, $\eta_{p}^{2}$=0.455; interaction: *F*(9,39) = 4.02, *p* < 0.001, $\eta_{p}^{2}$=0.481). The RMS values in the child group (10–15 years old) and adult group (35–40 years old) were significantly higher than those in most elderly groups (65–80 years old) in the biceps brachii. Figure 2D shows the relative change in RMS after fatigue in each age group. After fatigue, most age groups showed an increase in RMS. In the force decrease phase, the adult group (20–25 years old) exhibited a significantly higher relative change in RMS of the biceps brachii compared to most other age groups.

### 3.2 MU discharge characterization and MUAP morphology

**Figure 3:**
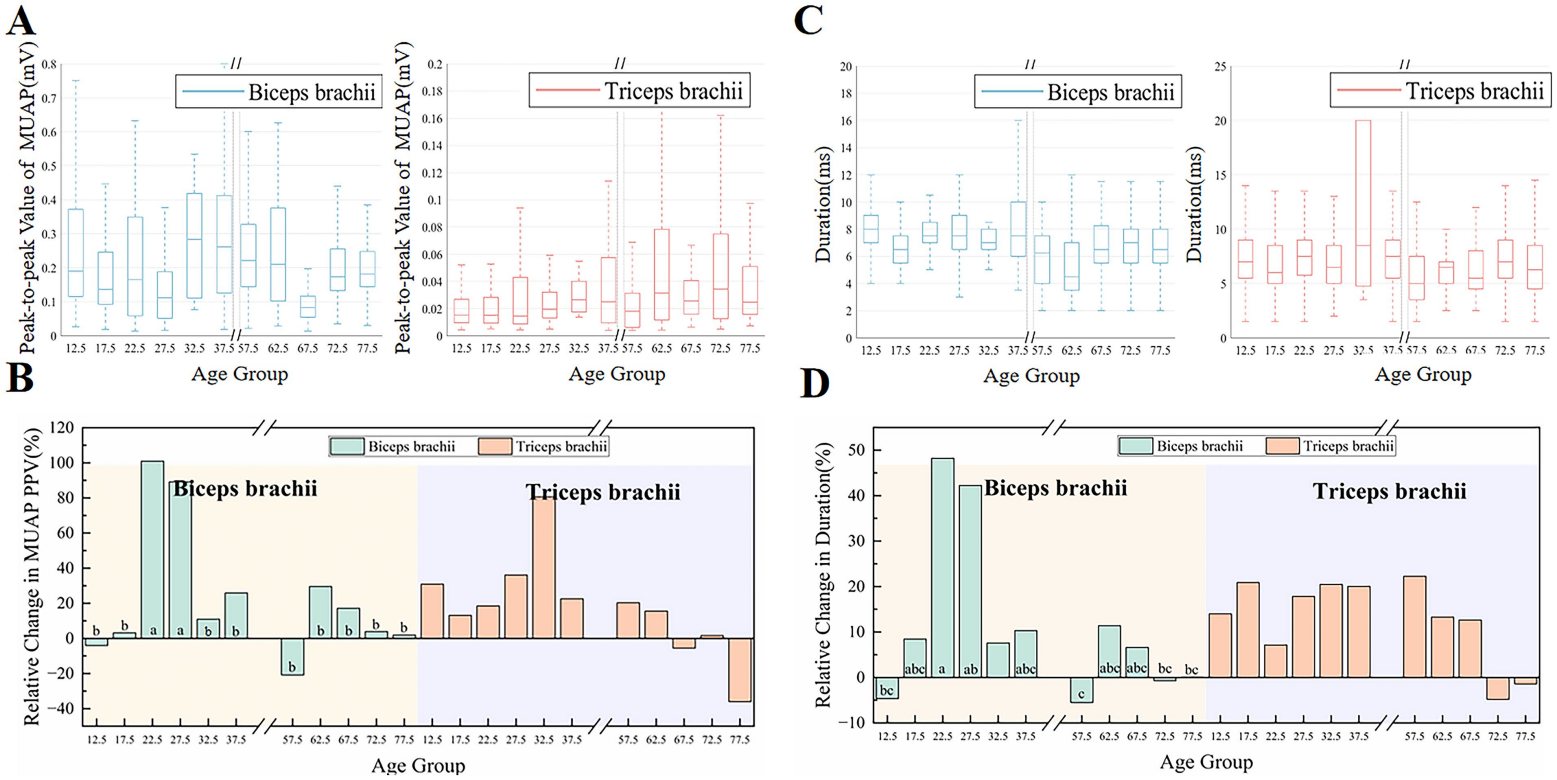
The changes in MUAP PPV and duration before and after fatigue across different age groups. **(A,C)** The peak-to-peak values and durations of MUs in the biceps and triceps across age groups before fatigue, while **(B,D)** show the relative changes in peak-to-peak values and durations after fatigue.

Figure 3 illustrates the morphological characteristics of MUs. Analysis revealed that MUAP PPV in the biceps brachii was significantly influenced by fatigue and the fatigue × age interaction, whereas the main effect of age alone was not significant (fatigue: *F*(1,34) = 12.26, *p* < 0.001, $\eta_{p}^{2}$=0.27; age: *F*(9,34) = 1.59, *p* = 0.157, $\eta_{p}^{2}$=0.30; interaction: *F*(9,34) = 4.24, *p* < 0.001, $\eta_{p}^{2}$=0.53). In contrast, MUAP duration was significantly influenced by fatigue, age, and their interaction for both the biceps brachii (fatigue: *F*(1,30) = 12.46, *p* < 0.001, $\eta_{p}^{2}$=0.29; age: *F*(9,30) = 3.85, *p* = 0.002, $\eta_{p}^{2}$=0.536; interaction: *F*(9,30) = 3.68, *p* = 0.003, $\eta_{p}^{2}$=0.525) and triceps brachii (fatigue: *F*(1,35) = 27.413, *p* < 0.001, $\eta_{p}^{2}$=0.439; age: *F*(9,35) = 2.771, *p* = 0.015, $\eta_{p}^{2}$=0.416; interaction: *F*(9,35) = 4.54, *p* < 0.001, $\eta_{p}^{2}$=0.54). As shown in Figure 3A, before fatigue, the MUAP PPV of the biceps brachii in the child group (15–20 years old) and the elderly group (65–80 years old) was lower than that in the adult group (35–40 years old). This phenomenon may be related to the smaller muscle fiber groups of the MUs or their more scattered spatial distribution. However, in the triceps brachii, the MUAP PPV values of the majority of the elderly group (60–80 years old) were significantly higher than those of the child group (10–20 years old) and the adult group (25–30 years old). These results suggest that the physiological changes in different muscle groups may vary with age. Regarding the duration of MUAP before fatigue (Figure 3C), the duration of the biceps brachii in the elderly group was significantly shorter than that in the child group (10–15 years old) and the adult group (20–40 years old). Simultaneously, the elderly group (55–60 years old) in the triceps brachii also showed a significantly shorter MUAP duration compared to the other age groups.

Figures 3B,D show the relative changes in the MUAP PPV and MUAP duration after fatigue. After fatigue, the PPV and duration of MUAPs in most age groups increased. The adult group (20–30 years old) exhibited the greatest increase in MUAP PPV and duration in the biceps brachii, which was significantly higher than in the other groups. There were no significant changes in the triceps brachii.

**Figure 4:**
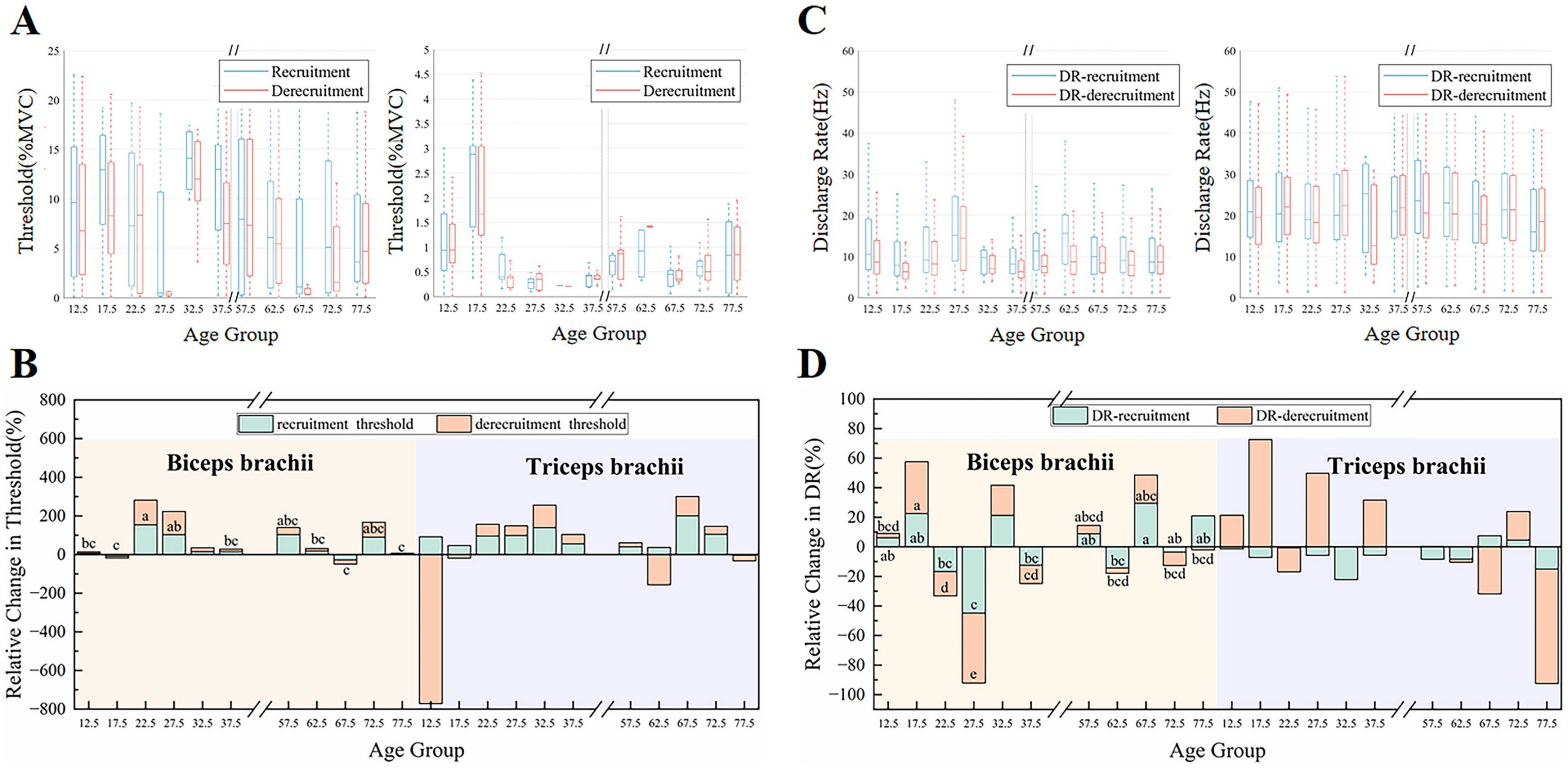
The changes in threshold and discharge rate before and after fatigue across different age groups. **(A,C)** Show the changes in recruitment and derecruitment thresholds, as well as discharge frequency of the biceps and triceps across age groups before fatigue. **(B,D)** Display the relative changes in recruitment and derecruitment thresholds, as well as discharge frequency, after fatigue.

Figure 4 presents the dynamic changes in recruitment and derecruitment thresholds, as well as discharge frequency, before and after fatigue across different age groups. Recruitment threshold was significantly influenced by fatigue, age, and their interaction in the biceps brachii (fatigue: *F*(1,36) = 5.33, *p* = 0.027, $\eta_{p}^{2}$=0.129; age: *F*(9,36) = 2.356, *p* = 0.033, $\eta_{p}^{2}$=0.371; interaction: *F*(9,36) = 3.986, *p* < 0.001, $\eta_{p}^{2}$=0.449). Derecruitment threshold in the biceps brachii was significantly influenced by age and the fatigue × age interaction, whereas the main effect of fatigue alone was not significant (fatigue: *F*(1,31) = 2.457, *p* = 0.127, $\eta_{p}^{2}$=0.073; age: *F*(9,31) = 4.244, *p* < 0.001, $\eta_{p}^{2}$=0.552; interaction: *F*(9,31) = 2.689, *p* = 0.019, $\eta_{p}^{2}$=0.438). The discharge rate was strongly influenced by the fatigue × age interaction, showing greater modulation during both recruitment (fatigue: *F*(1,30) = 0.766, *p* = 0.39, $\eta_{p}^{2}$=0.025; age: *F*(9,30) = 2.1, *p* = 0.065, $\eta_{p}^{2}$=0.383; interaction: *F*(9,30) = 3.04, *p* = 0.01, $\eta_{p}^{2}$=0.48) and derecruitment (fatigue: *F*(1,34) = 5.51, *p* = 0.025, $\eta_{p}^{2}$=0.139; age: *F*(9,34) = 1.813, *p* = 0.102, $\eta_{p}^{2}$=0.321; interaction: *F*(9,34) = 4.517, *p* < 0.001, $\eta_{p}^{2}$=0.545) in the biceps brachii. However, the main effects of fatigue and age were less pronounced during recruitment, whereas only the effect of fatigue reached significance during derecruitment.

As shown in the figure, the derecruitment threshold of most groups in the biceps brachii was lower than the recruitment threshold before fatigue (Figure 4A). The recruitment threshold of most elderly groups (55–80 years old) was significantly lower than that of most other age groups. Additionally, the discharge frequency during MU recruitment in the biceps brachii and triceps brachii was significantly lower in the elderly groups (55–80 years old) compared to the adult group (25–30 years old) (Figure 4C). The discharge frequency during MU recruitment in the biceps brachii of the child group (10–20 years old) was also significantly lower than that in the adult group (25–30 years old), further highlighting the impact of age on neuromuscular activation patterns.

The recruitment and derecruitment thresholds for the majority of age groups exhibited an increased trend following fatigue, as seen in Figure 4B. However, the child group’s changes following fatigue were less pronounced. In the biceps brachii, the change in derecruitment threshold after fatigue in the 20–25 age group was significantly higher than that in most elderly groups (60–80 years old). Figure 4D shows the relative changes in discharge frequency during recruitment and derecruitment after fatigue. With increasing age, the post-fatigue trends in each group differed. In the biceps brachii, most adult groups (20–40 years old) exhibited a decreasing trend in discharge frequency after fatigue, while most elderly groups (55–80 years old) and the child group (10–20 years old) showed an increasing trend in discharge frequency.

### 3.3 MUSTs common neural drive

**Figure 5:**
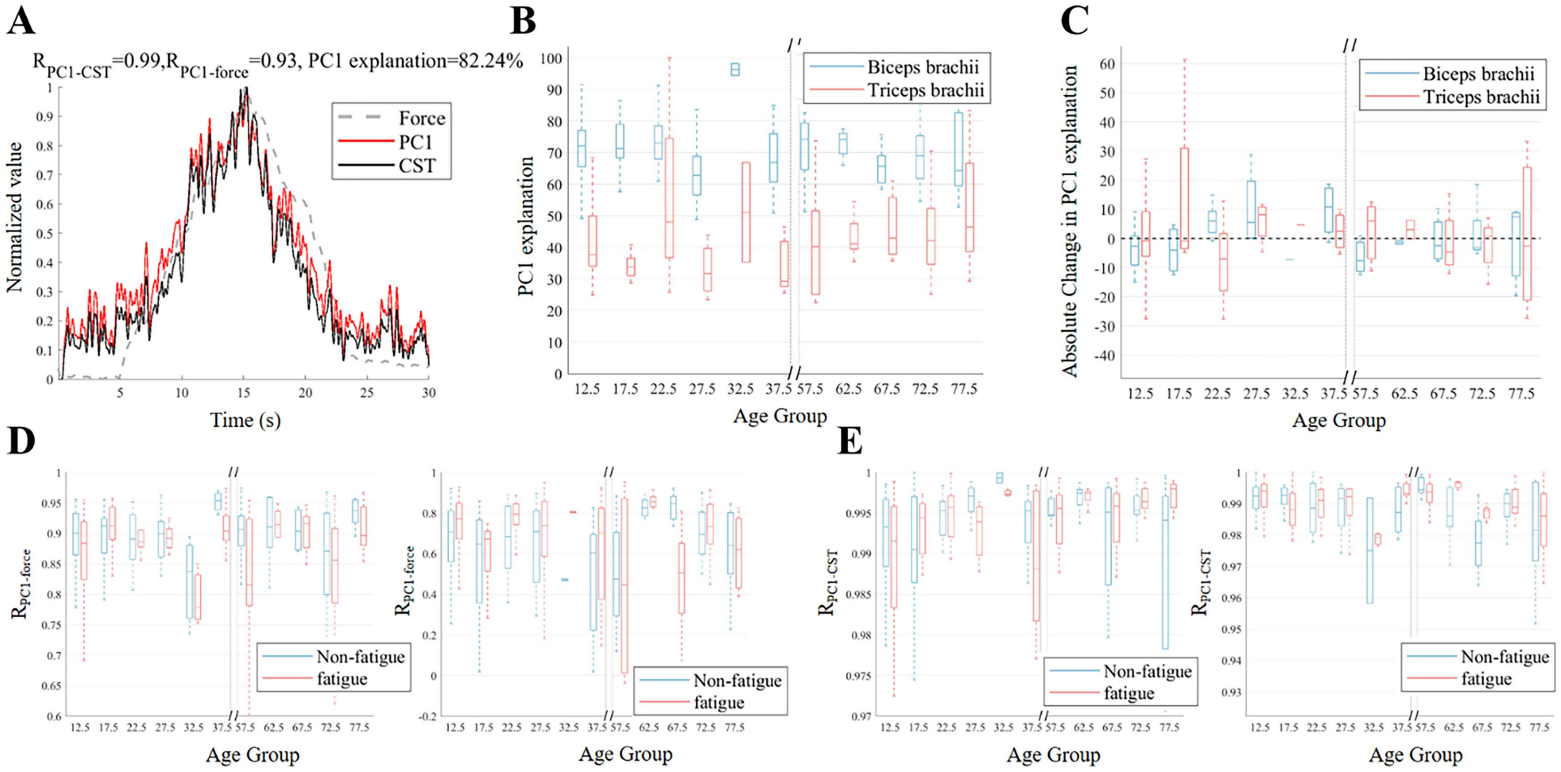
Changes in common input before and after fatigue across different age groups. **(A)** Illustrates example PC1, discharge rate of CST, and force signals. **(B,C)** The variance explanation of PC1 across age groups before fatigue and the absolute change in the variance explanation of PC1 after fatigue. **(D,E)** Display the correlation coefficients between PC1 and force, as well as the correlation between PC1 and CST, before and after fatigue for the biceps (left) and triceps (right).

Figure 5 presents the results of PCA on the MU discharge rate. In a typical example (Figure 5A), PC1 accounted for more than 80% of the variance and exhibited a high correlation with the strength curve and CST. For PC1-explained variance, the mixed 2 × 10 ANOVA showed no significant main or interaction effects (fatigue: *F*(1,32) = 0.26, *p* = 0.615, $\eta_{p}^{2}$=0.008; age: *F*(9,32) = 1.01, *p* = 0.454, $\eta_{p}^{2}$=0.221; interaction: *F*(9,32) = 1.80, *p* = 0.106, $\eta_{p}^{2}$=0.336). Figure 5B shows the variance explained by PC1 for the biceps brachii and triceps brachii in different age groups before fatigue. While the variance explained by PC1 in the biceps brachii of the elderly group (65–80 years old) was lower than that in the majority of adult groups (20–40 years old), it was higher in the child group (10–20 years old) than in the majority of adult groups (25–40 years old). Figure 5C presents the absolute changes in the variance explained by PC1 after fatigue. The median variance explained by PC1 in the child group (10–20 years old) and the elderly group (55–75 years old) showed a downward trend. In contrast, the median variance explained by PC1 in most adult groups (20–40 years old) showed an upward trend. Figure 5D shows the correlation between PC1 and muscle strength. Overall, the correlation in the biceps brachii was generally higher than that in the triceps brachii. Figure 5E displays the correlation between PC1 and CST. Both for the biceps brachii and triceps brachii, the correlation between PC1 and CST remained high before and after fatigue, with correlation coefficients greater than 0.96.

### 3.4 Main findings in results

The results of this study reveal significant differences in neuromuscular control mechanisms under fatigue across different age groups. In terms of force control, the adult group demonstrated the best tracking accuracy, while the child and elderly groups performed worse, with fatigue negatively impacting force control stability in all age groups. Time-frequency domain analysis of the EMG signals showed that MPF decreased in most age groups, and the RMS increased after fatigue. Furthermore, in-depth analysis of MU characteristics revealed differences in MUAP morphology between the child, elderly, and adult groups, with the adult group showing the most significant increase in PPV and duration of the MUAP after fatigue. After fatigue, adults exhibited decreased discharge rate with increased recruitment threshold, whereas children showed elevated discharge rate with minimal threshold change, and older adults displayed smaller increases in both parameters. Finally, the study of neural drive coordination indicated that fatigue weakened the stability of the nervous system’s coordination in both the child and elderly groups, making it harder for them to maintain common neural drive. In contrast, the adult group showed an enhanced ability to coordinate this drive in response to fatigue.

## 4 Discussion

### 4.1 Decomposition performance before and after fatigue

**Figure 6:**
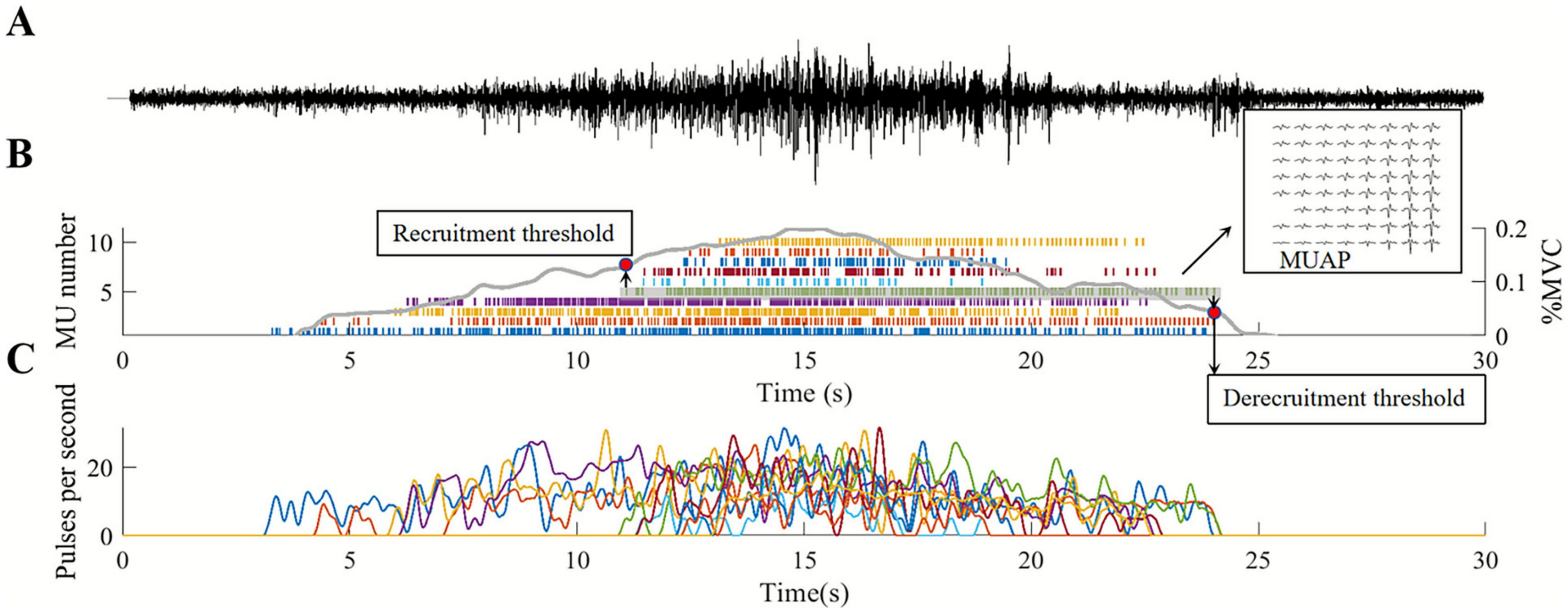
An example of decomposition results. **(A)** The sEMG signal example of the biceps from a subject before fatigue. The corresponding MUSTs decomposed from the sEMG are shown in **(B)**. Each vertical bar represents the discharge of a MU, with different colors indicating the discharge sequence of each MU. The gray line represents the force measured in this experiment. A multi-channel MUAP waveform example is shown in the subfigure. **(C)** Displays the discharge rate curve for each MU corresponding to the MUSTs in **(B)**.

Figure 6 presents a representative example of sEMG decomposition in a subject under a non-fatigue state. The decomposition identified 10 MUs, and the analysis of MUSTs and discharge frequency revealed the discharge patterns of these MUs. The force signal was synchronized with the sEMG signal, providing a clear correlation between muscle activity and force generation. Table 2 summarizes the decomposition of the biceps and triceps before and after fatigue in different age groups. As shown in the table, the biceps exhibited a significantly higher PNR than the triceps, suggesting better decomposition quality. The 30–35 age group had the fewest MUs identified, likely due to the smaller sample size and potential individual differences. Despite significant differences in the physiological characteristics and levels of fatigue among participants from different age groups, the number of MUs obtained from the decoding remained relatively consistent. This is mainly due to the inherent stability of the decomposition algorithm. It is crucial to note that the subsequent analyses in this study are based solely on the MUs that were successfully decoded.

**Table 2:** Decomposition across age groups before and after fatigue.

Age group	MU number (Biceps)		Pulse-to-noise ratio (dB)		MU number (Triceps)		Pulse-to-noise ratio (dB)	
	Non-fatigue	Fatigue	Non-fatigue	Fatigue	Non-fatigue	Fatigue	Non-fatigue	Fatigue
10–15	9 ± 5	9 ± 5	28 ± 4	28 ± 4	14 ± 7	17 ± 8	24 ± 3	25 ± 3
15–20	9 ± 5	10 ± 5	28 ± 4	28 ± 4	15 ± 4	14 ± 9	25 ± 3	25 ± 4
20–25	8 ± 3	8 ± 2	28 ± 4	30 ± 4	11 ± 9	14 ± 6	25 ± 4	25 ± 4
25–30	11 ± 3	8 ± 3	26 ± 4	28 ± 5	15 ± 2	12 ± 5	24 ± 3	25 ± 3
30–35	3 ± 1	3 ± 2	29 ± 3	34 ± 4	3 ± 4	7 ± 2	24 ± 3	26 ± 4
35–40	15 ± 3	11 ± 5	28 ± 4	29 ± 4	12 ± 5	17 ± 8	25 ± 3	25 ± 4
55–60	13 ± 3	12 ± 4	28 ± 4	28 ± 4	15 ± 5	14 ± 6	25 ± 3	25 ± 3
60–65	12 ± 3	14 ± 3	27 ± 4	27 ± 4	15 ± 2	20 ± 9	24 ± 3	24 ± 3
65–70	13 ± 6	14 ± 6	27 ± 4	26 ± 3	10 ± 5	9 ± 7	24 ± 3	24 ± 3
70–75	11 ± 5	10 ± 6	27 ± 4	29 ± 4	12 ± 7	15 ± 7	25 ± 3	25 ± 4
75–80	13 ± 6	13 ± 6	27 ± 4	27 ± 4	10 ± 5	7 ± 5	25 ± 4	25 ± 4

### 4.2 Effects of fatigue on force tracking and sEMG characteristics across ages

The study showed that age had a significant impact on the stability of muscle strength control. As shown in the figure, the elderly group exhibited the highest RMSE values, which may be due to a decline in their MU recruitment and derecruitment efficiency, leading to reduced muscle strength control precision (Verschueren et al., 2022). At the same time, the child group (10–20 years old) exhibited weaker neuromuscular coordination and slower response times than adults due to the incomplete development of their nervous system, leading to a relatively higher RMSE value. Under fatigue, the RMSE generally increased across all age groups, reflecting the impact of fatigue on the stability of muscle force output. This phenomenon was most pronounced in the 55–65 age group, likely related to muscle degeneration and reduced adaptability of the nervous system (Cardozo et al., 2013).

Furthermore, this study investigated the impact of age and fatigue on the time-frequency domain characteristics of sEMG signals. Significant main effects of fatigue and age, as well as a significant fatigue × age interaction, were observed for both MPF and RMS, indicating that fatigue and age jointly influenced the spectral and amplitude characteristics of sEMG signals. In the biceps brachii, the MPF during the force rising phase was higher in the adult group than in the falling phase, while the elderly group (55–80 years old) exhibited the opposite pattern. This difference may be due to age-related changes in neural compensation mechanisms. In young adults, the higher MPF during the force-rising phase reflects progressive recruitment of higher-threshold motor units together with an increase in muscle-fiber conduction velocity (Farina and Merletti, 2004; Park et al., 2016). In older adults, age-related neural compensation is characterized by increased low-frequency common synaptic input and reduced gamma-band strength, leading to a lower-frequency bias in the EMG spectrum and an alteration of the phase contrast in MPF (Castronovo et al., 2018). The MPF of the biceps brachii in the child group (10–15 years old) was significantly lower than that in the elderly group (55–80 years old), primarily because of the slower conduction velocity of muscle fibers in children, which is linked to the incomplete maturation of nerve myelination and the relatively higher proportion of Type I fibers. After fatigue, the MPF of the biceps and triceps brachii muscles decreased in most age groups, as muscle fiber membrane excitability changed and action potential conduction speed slowed. The decline was most pronounced in the 55–65 age group, possibly related to the reduced fatigue adaptation and decline in fast-twitch muscle fiber function in elderly adults (Tudoraşcu et al., 2014).

As people age, neuromuscular degeneration, a reduction in motor neurons, and slower nerve conduction, along with muscle atrophy and the loss of fast-twitch fibers, lead to lower muscle activation levels and weaker electromyographic signals in older adults. This is demonstrated by the fact that the RMS of the biceps brachii in children and adults was significantly higher than that of the elderly group before fatigue. Most age groups showed an increase in RMS values following fatigue, suggesting increased muscular activity during a state of fatigue. Adults showed the largest post-fatigue rise in RMS, likely because their compensation emphasized recruiting higher-threshold motor units and greater MUAP synchronization (McManus et al., 2015).

### 4.3 Effects of fatigue on discharge performance and MU morphology across ages

Studies of MUAP morphology have revealed the effects of age on fatigue adaptation and differences in recruitment patterns of different muscle groups. The results of the pre-fatigue study showed that the biceps brachii and triceps brachii muscles exhibited significantly different MUAP PPV across different age groups, suggesting that these two muscles may have distinct age-related mechanisms of neuroadaptive changes.

In the biceps brachii, MUAP PPV was significantly affected by fatigue and showed a robust fatigue × age interaction, whereas MUAP duration in both the biceps and triceps was significantly influenced by fatigue, age, and their interaction. The increase in MUAP PPV in the biceps brachii muscle after fatigue in most age groups may be due to the nervous system compensating for the lack of output by increasing MU recruitment. The significant increase in the 20–30 age group relative to the other groups suggested that adults have a greater capacity for adaptation and neural compensation in the face of fatigue (Mota et al., 2020). However, no significant differences were observed in the triceps brachii, possibly because the triceps brachii, as a muscle group that plays a stabilizing role in a wide range of daily activities, may have a relatively more stable recruitment of MUs (Wilson et al., 2015). The greatest rise in post-fatigue biceps brachii duration was observed in adults. Adults typically have a higher proportion of Type II fibers, which are more fatigue-prone. During sustained contractions, these fibers accumulate metabolites and develop ionic disturbances that slow conduction and repolarization, thereby prolonging MUAP duration (Valenčič et al., 2024). On the other hand, because of the fatigue resistant characteristics of Type I fibers, the MUAP duration increased only a little in the child and elderly groups with a higher percentage of these fibers.

The analysis of the recruitment threshold and derecruitment threshold further revealed the control strategies of MUs. The phenomenon where the derecruitment threshold was lower than the recruitment threshold was typically closely related to fatigue and neuroadaptive adjustments (Avrillon et al., 2024; Blazevich et al., 2024). A key finding emerging from our data is that the recruitment threshold was modulated by fatigue, age, and their interaction in the biceps brachii. Regarding firing behavior, the MU discharge rate was primarily driven by the Fatigue × Age interaction in both recruitment and derecruitment phases. During recruitment, the main effect of fatigue was non-significant, whereas during derecruitment, the main effect of age did not reach significance. The lower recruitment thresholds and discharge frequencies in the elderly group may be due to the fact that, with aging, Type II fibers gradually decrease, while Type I fibers relatively increase (Habenicht et al., 2020). The discharge frequency of the adult group was higher than that of the child group, reflecting that the ability to recruit MUs and the degree of synchronization of MU firing gradually improve during development (Del Vecchio et al., 2020b). After fatigue, the discharge frequency of the biceps brachii in the 20–25 age group decreased, but the increase in their recruitment threshold was significantly greater than that observed in the other groups. This suggested that, although fatigue prevented maintaining a high discharge frequency, adults were still able to maintain relatively stable force output under fatigue by recruiting higher-threshold MUs (Valenčič et al., 2024). According to the study’s findings, children may have depended more on firing frequency modulation during exhaustion because of their reduced capacity to recruit MUs and lower levels of central activation (Bontemps et al., 2019; Miller et al., 2019). In contrast, the elderly showed smaller changes in both recruitment threshold and discharge frequency after fatigue, indicating that their neuromuscular system had a weaker ability to adapt to fatigue (Roos et al., 1997).

### 4.4 Common neural control and synergy

In the present investigation, PCA elucidated the coordinated regulatory patterns of MU discharge rates across age cohorts and fatigue conditions. The higher variance of PC1 explanation in the biceps brachii muscle in the child group compared to the adult group may be due to the fact that children’s central nervous systems are still developing and depend more on cortical mechanisms to regulate antagonist muscle activity during isometric contractions (Figure 5B). Although the ANOVA results did not reach statistical significance, the observed trend and moderate effect size ($\eta_{p}^{2}$= 0.336) suggest that the changes in the Fatigue × Age interaction were not random but represent a systematic modulation of the common neural drive. Compared to the adult age group, the PC1 explained variance in the biceps brachii was considerably lower in the 65–80 age group. Age-related declines in MU synchronization and more scattered neural drive patterns were most likely the causes of this discrepancy (Erim et al., 1999). Figure 5C further revealed the impact of fatigue on this coordination. Both the child group and the elderly group showed a decrease in the PC1 explained variance after fatigue, suggesting that individuals with incomplete development or aging had more difficulty maintaining consistent drive under fatigue (Dos Santos et al., 2020; Woods et al., 2022). However, the adult group showed an increase in the PC1 explained variance after fatigue, possibly reflecting a stronger coordination in coping with fatigue at this age stage (McManus et al., 2016). The higher correlation between PC1 and muscle strength in the biceps brachii compared to the triceps brachii may be due to the biceps brachii’s stronger neural drive and greater involvement in task performance, as it is more closely linked to cortico-spinal activity and muscle synergy coordination (Ortega-Auriol et al., 2023; Butler et al., 2025). Nonetheless, the strong correlations between PC1 and CST across all groups indicate that, despite variations in synergy due to fatigue and age, the essential neural mechanisms underlying force generation remain preserved to a substantial degree.

### 4.5 Limitations and future directions

Although the present study provides insight into the effects of fatigue and aging on neuromuscular control mechanisms, several limitations remain. First, we used a triangular isometric force trajectory at a fixed target force (20% MVC) with a relatively slow rate of force development (2% MVC/s). Because the rate of force development can shape recruitment and firing strategies, some age-group differences may reflect this specific task rather than a general property across tasks. Future studies should test multiple rates of force development, plateau paradigms, and a range of target-force levels, and validate the findings across different movement patterns (Klass et al., 2008). Second, the behavior of all MUs, particularly the electrical activity of the deep muscles, cannot be completely captured by the high-density sEMG approach, particularly in older adults with muscular atrophy. Although data for the triceps brachii were collected, we did not perform an in-depth analysis of its motor unit properties, which limits our comprehensive understanding of the role of antagonist muscle modulation under conditions of fatigue and aging. Moreover, endurance time and other indices of performance fatigability were not analyzed in detail in the present study, and future work should incorporate these measures to better contextualize age-related differences in motor unit behavior. Third, we did not perform an *a priori* power analysis. The total sample size was determined based on field precedent, available resources, and the feasibility of data collection. However, to enhance the rigor and reproducibility of future studies, we will conduct a priori power analyses to prospectively justify the sample size, ensuring that the study is adequately powered to detect effect sizes of interest. Finally, based on prior research, sex differences can affect MU recruitment strategies, discharge rates, and neuromuscular responses to fatigue, owing to divergences in muscle-fiber composition, hormonal milieu, and neural drive (Lulic-Kuryllo and Inglis, 2022; Inglis and Cabral, 2025). Because our primary objective was to characterize age-related adaptations to fatigue, we did not perform explicit within-cohort analyses of sex effects, which we acknowledge as a limitation. To improve robustness and account for sex-specific effects, future studies should recruit sex-balanced cohorts and conduct adequately powered analyses of motor-unit behavior in males and females.

## 5 Conclusion

This study systematically investigated the impact of age on neuromuscular control ability under conditions of fatigue by decomposing high-density sEMG signals into MUSTs using the CKC algorithm. The results showed significant differences across age groups in force tracking performance, sEMG signal characteristics, and MU behavior. Specifically, the adult group demonstrated the best force tracking performance, while the child and elderly groups performed poorly. This may be attributed to the reduced adaptability of the neuromuscular system and decreased MU recruitment efficiency in older adults. Under fatigue, the RMSE values increased in all age groups, indicating the universal negative impact of fatigue on motor control accuracy.

Additionally, sEMG signal analysis showed that the elderly group’s MPF decreased more significantly after fatigue, but changes in RMS were comparatively small. This suggested that their neuromuscular system’s compensatory capacity was limited. An examination of MU discharge characteristics revealed that the adult group tended to engage higher-threshold MUs to compensate for the strength decline induced by fatigue, while the elderly group showed less change in recruitment thresholds and discharge rates, indicating reduced neural flexibility. The child group may have relied more on frequency modulation after fatigue because they displayed smaller variations in recruitment threshold. PCA results further indicated that maintaining a steady neural drive becomes more challenging with aging or incomplete neural development.

Mechanistically, the age-related differences observed in this study likely arise from a combined deterioration of peripheral and central neuromuscular processes. With aging, progressive muscle fiber loss and collateral reinnervation result in enlarged but less finely controlled motor units, reducing the peripheral contractile reserve available during fatigue. In parallel, declines in motoneuron excitability, synaptic efficacy, and corticospinal drive diminish the central nervous system’s capacity to flexibly adjust recruitment and rate-coding strategies when force begins to fail. In contrast, children operate with an incompletely mature motor system, characterized by less refined corticospinal connectivity, reduced myelination, and suboptimal intermuscular coordination, which limits their ability to generate and stabilize precise neural drive under fatigue. Together, these peripheral and central constraints restrict the ability of both developing and aging neuromuscular systems to reorganize activation patterns in response to fatigue, whereas adults retain a broader functional reserve that supports more adaptive motor-unit reconfiguration. This study provides important experimental evidence for understanding age-related neuromuscular dysfunction and lays a theoretical foundation for the development of targeted rehabilitation strategies. Additionally, our framework can be further extended to investigate neuromuscular alterations in pathological populations, while the age-dependent motor unit patterns identified in this study provide an important physiological reference for neurological and neuromuscular healthcare.

## Data Availability

The raw data supporting the conclusions of this article will be made available by the authors, without undue reservation.
