# Supplementary material for: Effects of fatigue on motor unit characteristics in isometric elbow contractions across age groups
Source: Front Neurosci. 2026 Jan 5;19:1747360. doi: 10.3389/fnins.2025.1747360 (PMC12812920; doi:10.3389/fnins.2025.1747360)
Supplement: Supplementary file 1 [file Table_1.DOCX]

Supplementary Material

# Supplementary Tables

We have included an additional table in the attachment to provide more information about the participants. In the sex column, “1” indicates male, and “0” indicates female.

| **subject** | **sex** | **age** | **BMI** | **weight（**kg） | **height（**cm） | **maximum force（**kg） |
| --- | --- | --- | --- | --- | --- | --- |
| 1 | 1 | 13 | 14.49 | 28 | 139 | 7.3 |
| 2 | 0 | 72 | 20.78 | 48 | 152 | 10 |
| 3 | 1 | 12 | 15.73 | 34 | 147 | 7.5 |
| 4 | 1 | 14 | 19.38 | 56 | 170 | 15.1 |
| 5 | 1 | 12 | 16.42 | 41 | 158 | 9.2 |
| 6 | 0 | 77 | 22.35 | 53 | 154 | 8 |
| 7 | 0 | 11 | 16.80 | 32 | 138 | 7.2 |
| 8 | 0 | 11 | 19.29 | 40 | 144 | 6 |
| 9 | 0 | 12 | 18.30 | 39 | 146 | 7.4 |
| 10 | 1 | 75 | 22.39 | 67 | 173 | 24 |
| 11 | 1 | 72 | 25.40 | 70 | 166 | 20 |
| 12 | 1 | 70 | 23.14 | 63 | 165 | 20.8 |
| 13 | 1 | 74 | 23.45 | 71 | 174 | 18.6 |
| 14 | 1 | 61 | 24.30 | 63 | 161 | 17.2 |
| 15 | 1 | 72 | 26.26 | 75 | 169 | 20.5 |
| 16 | 1 | 66 | 24.44 | 61 | 158 | 11.6 |
| 17 | 1 | 75 | 22.89 | 55 | 155 | 11 |
| 18 | 0 | 74 | 18.37 | 43 | 153 | 10.8 |
| 19 | 0 | 65 | 24.35 | 57 | 153 | 9.8 |
| 20 | 1 | 69 | 21.83 | 58 | 163 | 16.2 |
| 21 | 1 | 11 | 15.16 | 31 | 143 | 7 |
| 22 | 1 | 58 | 29.74 | 80 | 164 | 32 |
| 23 | 1 | 36 | 22.04 | 67.5 | 175 | 28.8 |
| 24 | 0 | 60 | 28.16 | 73 | 161 | 16.4 |
| 25 | 1 | 58 | 18.50 | 56 | 174 | 19.8 |
| 26 | 0 | 22 | 20.57 | 56 | 165 | 15.8 |
| 27 | 0 | 16 | 20.55 | 58 | 168 | 12.8 |
| 28 | 1 | 22 | 29.06 | 83 | 169 | 21.8 |
| 29 | 0 | 59 | 30.46 | 77 | 159 | 12.8 |
| 30 | 0 | 16 | 18.20 | 46 | 159 | 10.2 |
| 31 | 1 | 18 | 24.97 | 80 | 179 | 23.2 |
| 32 | 0 | 19 | 19.48 | 45 | 152 | 9.6 |
| 33 | 1 | 21 | 21.14 | 64 | 174 | 20.5 |
| 34 | 1 | 14 | 26.73 | 80 | 173 | 18 |
| 35 | 0 | 13 | 18.96 | 51 | 164 | 12.3 |
| 36 | 1 | 10 | 15.52 | 34 | 148 | 6.7 |
| 37 | 0 | 14 | 17.91 | 47 | 162 | 12.6 |
| 38 | 1 | 11 | 15.40 | 31.5 | 143 | 6.2 |
| 39 | 0 | 28 | 24.72 | 62.5 | 159 | 13.7 |
| 40 | 1 | 30 | 36.39 | 114 | 177 | 34.8 |
| 41 | 1 | 28 | 32.24 | 101 | 177 | 32.3 |
| 42 | 1 | 28 | 31.74 | 95 | 173 | 33 |
| 43 | 0 | 65 | 32.05 | 77 | 155 | 14.5 |
| 44 | 0 | 35 | 22.06 | 53 | 155 | 14 |
| 45 | 1 | 21 | 25.25 | 80 | 178 | 24.8 |
| 46 | 1 | 21 | 29.41 | 85 | 170 | 19.8 |
| 47 | 1 | 36 | 26.30 | 76 | 170 | 26.1 |
| 48 | 0 | 38 | 20.45 | 55 | 164 | 17 |
| 49 | 0 | 70 | 21.48 | 55 | 160 | 10.7 |
| 50 | 0 | 27 | 18.82 | 50 | 163 | 11.7 |
